# Reviewing manuscripts for scientific journals: recommendations for early career scientists

**DOI:** 10.1186/s13104-024-07060-8

**Published:** 2025-01-16

**Authors:** Diego A. Forero, Stephen J. Glatt, Marilyn H. Oermann

**Affiliations:** 1 School of Heath and Sport Sciences, Fundación Universitaria del Área Andina, Bogotá, Colombia; 2 Department of Psychiatry and Behavioral Sciences, SUNY Upstate Medical University, Syracuse, NY United States of America; 3 Duke University School of Nursing, Durham, NC United States of America

**Keywords:** Peer review, Scientific publishing, Life sciences, Health sciences

## Abstract

Publication of articles in international scientific journals has been one of the main strategies for the communication of scientific findings and ideas. Prepublication peer review is a fundamental aspect of the publishing process in indexed scientific journals and, associated with the large growth in journals and articles, there has been a recent challenge in having adequate peer reviewers for international journals. In this article, we provide a short overview of the publishing process, give recommendations to early career researchers about writing peer reviews of adequate quality, and discuss some possibilities for the future.

## Introduction

Publication of articles in international scientific journals has been one of the main strategies for the communication of scientific findings and ideas [1]. Prepublication peer review is a fundamental aspect of the publishing process in indexed scientific journals [2]. Peer review has been used by established journals throughout recent history as a standard for deciding on articles to publish, although the process and practices of peer review have evolved over the years [3].

A recent article estimated that the time dedicated to peer review during 2020 was more than 100 million hours, with an predicted cost of around 2.5 billion USD (based on an estimation of 6 h dedicated to each review) for reviewers from the US, UK and China [4]. Associated with the large growth in journals and articles [3], there has been a recent challenge in having adequate peer reviewers for international journals [5]. For example, a recent analysis of 19 international journals showed that over 180.000 reviewers were invited and that more than 113.000 of them did not accept the invitation to review [6].

In this article, we provide a short overview of the publishing process, give recommendations to early career researchers about writing peer reviews of adequate quality, and discuss some possibilities for the future of scientific publishing and peer review. These aspects are based on a review of the recent related literature [7–9] and the experience of the authors of this article as editors and peer reviewers for multiple journals.

## A brief introduction to the current peer review system in scientific journals

In the current scientific publishing environment in the health and life sciences, it is common that the authors submit their manuscript to a journal using an online platform [10]. After submission, a screening is carried out by staff from the publisher and the submitted manuscript is reviewed by an editor of the journal (an Editor-in-Chief or an Associate Editor) to decide whether the manuscript should be sent to peer reviewers or rejected prior to peer review, often referred to as desk rejection. In general, the higher the impact factor of the journal, usually signifying a larger number of submissions of manuscripts, the higher the rate of desk rejection [11].

If an editor considers that the manuscript is appropriate for the journal and its readers, and is ready to be sent to peer review, multiple potential reviewers are invited via an online platform. It is common that the editor sends multiple rounds of invitations to several potential reviewers. This is one of the major challenges, as many scientists do not accept, or do not answer, the invitations to be peer reviewers [5]. Although the number of needed reviews varies, it is quite common to have two independent peer reviews for each manuscript and the time allowed for each reviewer varies, but it is commonly between 2 and 4 weeks.

**Fig. 1:**
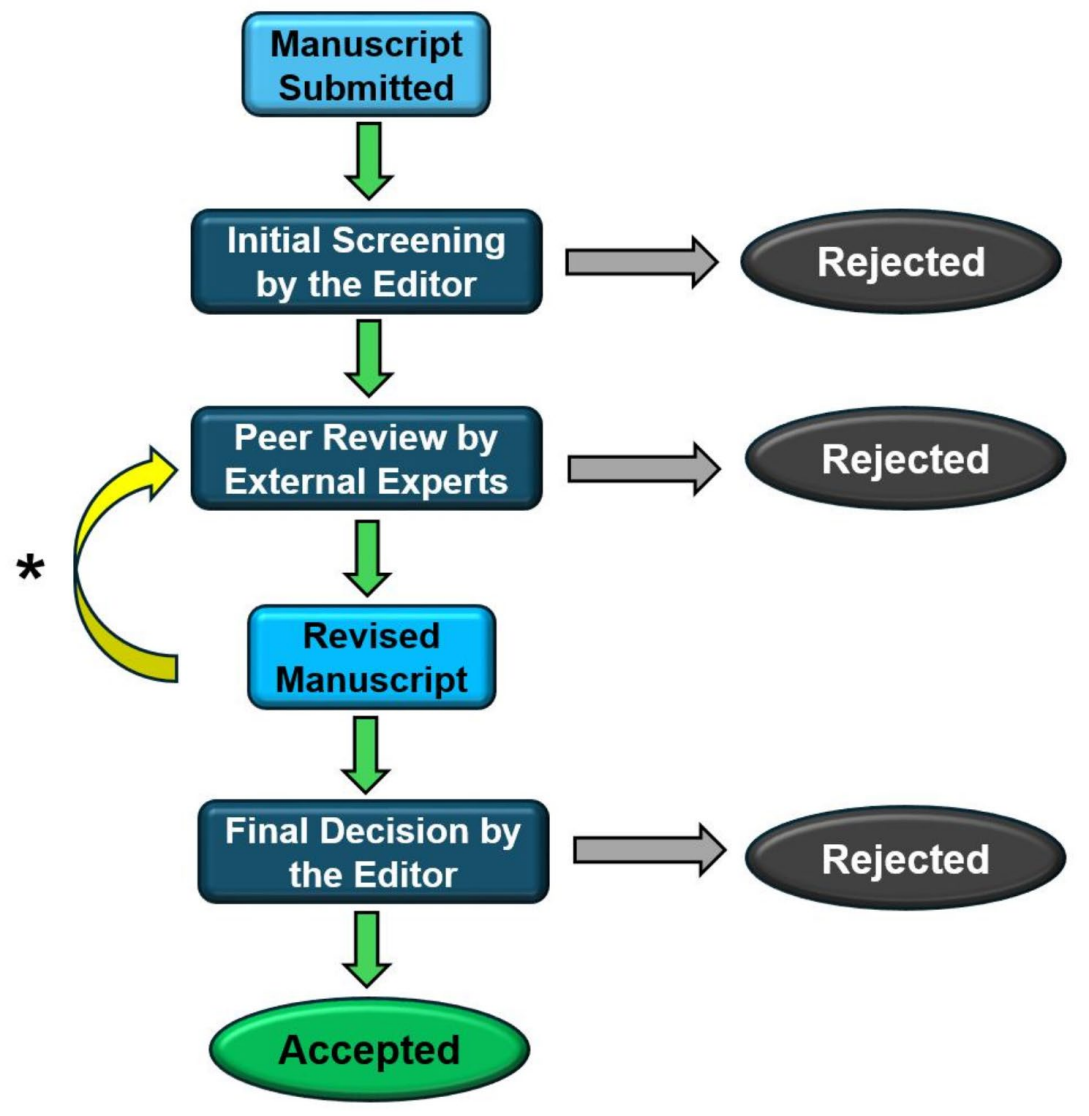
A simple overview of the general pre-publication peer review process carried out in scientific journals. * Note: In many cases, there are multiple review rounds

After the reviewers complete their review, assessing the accuracy, quality, and relevance of the manuscript for the journal, the editor makes an editorial decision with two main options: giving the opportunity to authors for addressing the comments of the reviewers or rejecting the manuscript [12]. In the case of the first option, the reviews are sent to the authors and, in a defined period, they resubmit a revised manuscript. When the revised manuscript is resubmitted, it is common that the editor sends it to the previous reviewers, who review it again. In some cases, a manuscript might have several rounds of review. After the peer review, the manuscript is accepted or rejected, a decision made by the editor, considering the comments of the reviewers. An overview of this process is depicted in Fig. 1.

**Fig. 2:**
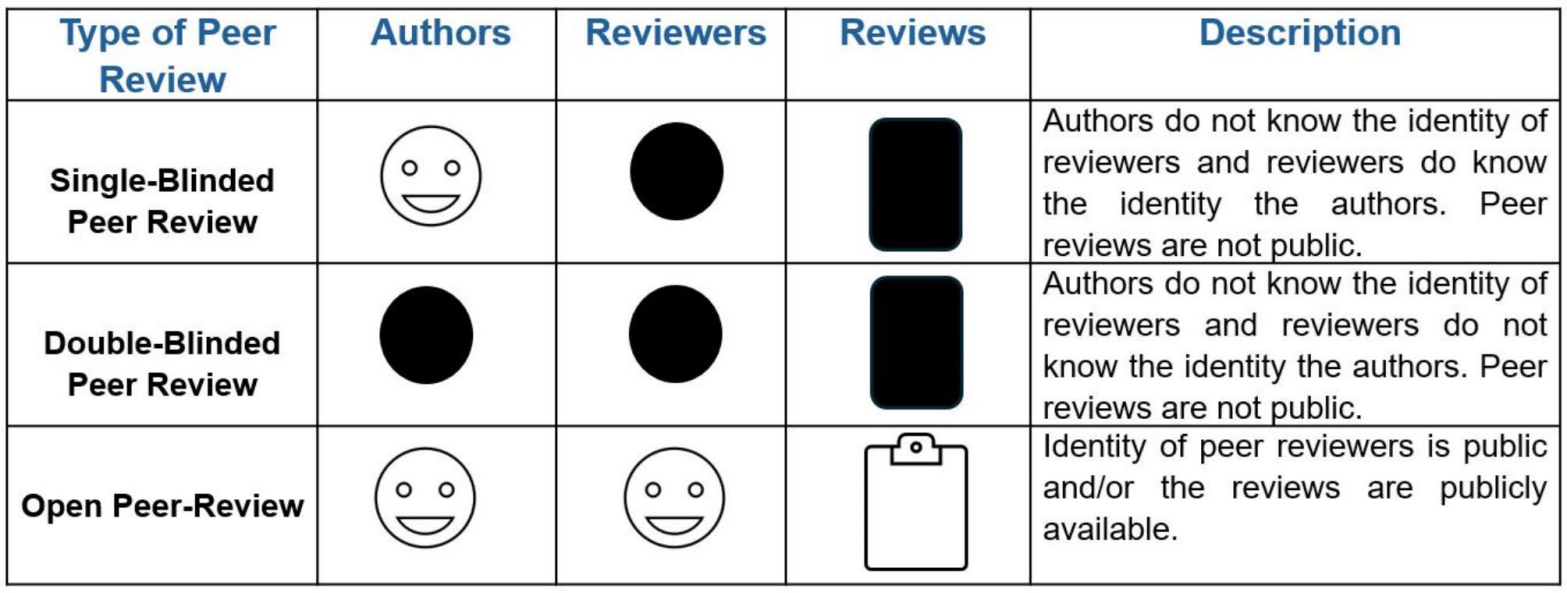
A brief description of the main types of pre-publication peer review carried out in scientific journals

Currently, there are three main types of pre-publication peer review: Single-blinded (where the authors do not know the reviewers but the reviewers know the authors); double-blinded (when neither authors nor reviewers know each other); and open (where authors and reviewers are known and/or the review is made available) [13] (Fig. 2). Additionally, there is the possibility for triple-blinded peer review, in which the editor does not know the identity of the authors or of the reviewers. Each of these types of reviews has advantages and disadvantages, from the perspective of the authors and reviewers [14]. In terms of open science, open peer reviews bring more transparency [14].

## The importance of being peer reviewer for scientific journals

There are multiple potential advantages for early career scientists serving as peer reviewers for international journals [9]. Some of these advantages include being exposed to the latest findings of the related research fields, contributing to improving the scientific literature, and learning about the publishing process from other perspectives, among others [7]. Being a peer reviewer is an integral part of the scientific publishing process, and it is difficult to think of a scenario where scientists are only authors [9]. Considering the advantages of being a peer reviewer, it is important for more early career researchers to serve as peer reviewers of international journals [4]: experience as a peer reviewer helps early career researchers to learn more about the different aspects of scientific publishing, particularly in their specific research fields, and to gain more visibility among editors of journals, among others.

## Key recommendations for early career scientists for reviewing manuscripts

In this section, we provide several useful suggestions about carrying out the peer review of a manuscript. Further detailed information of importance for early career scientists is available in existing training resources for peer reviewers (Table 1).

**Table 1:** Selected available training resources about peer review for early-career scientists

Resource	Link
Ethical guidelines for peer reviewers, Committee on Publication Ethics (COPE)	publicationethics.org/resources/guidelines/cope-ethical-guidelines-peer-reviewers
Step by step guide to reviewing a manuscript, Wiley	authorservices.wiley.com/Reviewers/journal-reviewers/how-to-perform-a-peer-review/step-by-step-guide-to-reviewing-a-manuscript.html
How to conduct a review, Elsevier	www.elsevier.com/reviewer/how-to-review
How to peer review, Springer Nature	www.springernature.com/la/authors/campaigns/how-to-peer-review
How to Write a Peer Review, PLOS	plos.org/resource/how-to-write-a-peer-review/

### Quality is the most important aspect in a peer review

The most important contribution of peer reviewers to the publishing process is to assess the quality of the research reported in the manuscript and the manuscript itself [2]. One of the main ways to evaluate the quality of a submitted manuscript is to carry out a detailed review of its adherence to common standards for the specific field, which involves several aspects such as study design, sample size, comparison groups and use of validated instruments, among others [15]. Reviewers are selected on the basis of their expertise in the methods or topics of the submitted manuscript. In addition, peer reviewers need to complete high-quality reviews and be willing to dedicate time to carry out a detailed evaluation of the submitted manuscript. A high-quality peer review usually involves a careful review of the different sections of the manuscript [9] (such as Introduction, Methods, Results, including tables and figures, Discussion, and References), in addition to supplementary files.

### Timely reviews are also key

In addition to providing high-quality peer reviews and reports about the methods and content of the manuscript, completing timely reviews is of particular importance for the authors of the submitted manuscripts. This means that reviewers should only accept invitations to review when they are sure of having the time to assess the manuscript in the proposed timeline. This also means that reviewers should decline an invitation to review when they do not have the time and to communicate this promptly to the journal [9].

### Take into account potential conflicts of interest

When accepting an invitation, the potential reviewer should take into account potential conflicts of interest, such as being a recent collaborator of an author of the manuscript. For single blinded and open peer reviews, the author names are known by the reviewer. In those cases, the potential reviewer should decline the invitation. Other types of competing interests, such as financial conflicts of interests [16], also should be carefully considered by the reviewer and reported to the editor.

### Take into account the type of manuscript you are reviewing

Similar to authors writing different types of manuscripts, such as research reports or literature reviews [10, 17], reviewing a research report is not the same as assessing a systematic review. Each type of manuscript has its own standards for peer review. For example, the contents and implications of a meta-analysis are different from randomized clinical trials of pharmacological interventions or a case report. Reporting guidelines are useful for reviewing manuscripts. Some examples of commonly used reporting guidelines are: PRISMA 2020 for systematic reviews and meta-analyses [18], CONSORT 2010 for randomized clinical trials [19], STROBE for observational studies [20] and STARD 2015 for diagnostic accuracy studies [21], among others. Consequently, the time needed to review each type of manuscript varies, and the key points to focus on during the review are also different [9].

### Differentiate between major and minor comments

In general, there are two main types of comments: major, which usually involves substantial methodological aspects, and minor, which usually involve aspects such as presentation or reporting of methods or results. It is important that reviewers highlight which are major comments, as usually these would involve a larger investment in time and efforts from the authors (and in some cases, doing additional experiments or redoing analyses). Minor comments, in contrast, often involve suggestions about presentation of figures and tables or about the writing in English.

### Provide valuable and constructive comments

It is important that you provide specific comments, ideally including the location of the respective issues in the manuscript [9]. Reviewers should take into account that both authors and reviewers are scientists. Reviewers should provide feedback to authors about issues with the manuscript and suggested revisions as well as positive feedback on the strengths of the study and manuscript. It is important that reviewers carefully write their reviews to avoid the use of derogatory terms. Vague or unspecific comments are not valuable for authors or for the editors.

### You are a peer reviewer, you are not a coauthor

A possible barrier for some novice reviewers is viewing the process as a time-consuming task. However, a different view is to see the process as providing comments, from the perspective of a peer reviewer, instead of giving long proposals of solutions to issues in the manuscript (a task for the authors, not for the reviewers). The benefits of being a peer reviewer outweigh the time commitment.

### You do not need to be an expert on everything reported in the manuscript

Do not worry if you are not an expert on the entire manuscript; this is one of the reasons there are several peer reviewers for each submitted manuscript. In cases of topics or research methodologies that are outside of your expertise, and need the perspective from another reviewer, you can highlight this to the editor in your peer report.

As a basic example for novice reviewers, the components of a hypothetical peer review report are presented in Table 2. In addition, taking advantage of developments in open science, we have consolidated available examples of open peer reviews for different types of articles (Table 3). Finally, taking into account the growth in post-publication verification of information presented in articles, we also consolidated examples of different reasons for retraction of published articles (Table 4). Post-retraction reviews would be valuable as training examples, for early career scientists, of major issues that were not detected in the pre-publication review of those articles.

**Table 2:** Anatomy of a (hypothetical) peer review report

Introduction	The manuscript by Smith et al. reports the results from a project focused on an intervention for improving mental health. Although the work is interesting, several major and minor issues should be addressed by the authors.
*Major Issues*	-The authors might consider increasing their sample size as the number of patients is relatively low.
*Minor Issues*	The rationale of the study should be presented more clearly in the Methods section. -Details of the statistical software used should be included in the Methods section -The presentation of Table 1 is not clear (column names are not indicative enough) and should be improved. -Several references in the Discussion section are outdated and should be updated.

**Table 3:** Examples of existing open peer reviews for different types of articles

Type of Article	Link for Open Review
Systematic review and meta-analysis	bmjopen.bmj.com/content/bmjopen/8/2/e018557.reviewer-comments.pdf
Cohort study	https://journals.plos.org/plosmedicine/article/peerReview?id=10.1371/journal.pmed.1003911
Case-Control study	https://link.springer.com/article/10.1186/s12916-021-01907-8/peer-review
Case report	https://journals.plos.org/plosntds/article/peerReview?id=10.1371/journal.pntd.0011647
Animal model	elifesciences.org/articles/39658/peer-reviews#content
Cell model	elifesciences.org/articles/31098/peer-reviews#content
Narrative review	https://journals.plos.org/digitalhealth/article/peerReview?id=10.1371/journal.pdig.0000082

**Table 4:** Examples of major scientific quality and reporting issues in retracted articles

Retracted article	Reasons for retraction
PMID: 38437195	*“****Concerns were raised about potential undisclosed use of an artificial intelligence tool****to generate text in the article due to inclusion of the phrase “regenerate response” and extensive reference list concerns. (****The Journal) was unable to verify 18 of the 76 cited references***, *and 6 additional references appear to contain errors.”* 10.1371/journal.pone.0302484
PMID: 21173882	“*The****authors did not provide the required IRB and informed consent information****relating to this study and it was determined the study did not meet the standard ethical publication requirements for studies involving human subjects in research*.” 10.2147/NDT.S296320
PMID: 32438138	*“After post-publication investigation*,* issues related to the following were identified in the article*: ***Inconsistency in study completion dates***,*** Unfeasible data***,*** Statistical errors****” DOI*: 10.1016/j.jogoh.2024.102820
PMID: 35351197	*“After publication*, ***concerns were raised regarding the statistical analysis in the article***. *Specifically*,* it is stated that the authors used the “Begger’s test”*,* which is not described in the literature”.* 10.1186/s13102-024-00994-3
PMID: 29523223	*“Following the publication*, ***concerns have been raised about a number of figures in this article***. *The western blots in this article were presented with atypical*,* unusually shaped and possibly anomalous protein bands in many cases.”* 10.32604/or.2024.055036
PMID: 37933361	***“The images presented in*** Fig. 1***have been plagiarized from an online lecture****that was uploaded to the internet*,* but removed by the original author after discovery of the plagiarism.” DOI*: 10.7759/cureus.r147
10.1007/s10876-020-01935-z	*“Recurring problems include*,* but are not limited to*, ***citations which do not support claims made in the text***,*** non-standard phrasing***, *anomalies in the figures and discrepancies in ethics approval statements.” DOI*: 10.1007/s10876-024-02558-4
PMID: 38482071	*“Following publication*,* the Editorial Office was made aware that****an undeclared conflict of interest existed for this manuscript***. *This lack of declaration was an oversight by the authors but impacted the editors’ fair assessment of the manuscript as well as the peer review process.”* 10.3389/fpsyt.2024.1292432

## Current challenges and needs in the peer review process

Although, in general, identification of plagiarism has been improved by automated tools [22], other recent aspects remain as challenges in terms of the verification of research integrity [23, 24]: tortured phrases (a term used to describe strange ways to write common terms, in order to avoid plagiarism detection) [25], image manipulation, and other types of data manipulation or fabrication [26]. Recently, there is a growing concern about the best ways to identify in submitted manuscripts text and images created by generative artificial intelligence tools [27].

In addition, paper mills, organizations that prepare and sell manuscripts to authors [28], are another major current challenge for peer review, particularly in some specific fields. Currently, identification of inadequate practices of authorship, such as gift or ghost authorship [29], also remains a challenge. Another concern, very recently, are review mills [30] that conduct fake peer reviews or manipulate them. In this context, there also has been recent criticisms about some indexed journals that might conduct, for different reasons, less rigorous peer reviews [28].

Currently, there is a major need related to peer review: training of young scientists in how to carry out a peer review of high quality [31]. Three main strategies might be implemented: creating modules about peer review in courses taught at MSc and/or PhD programs, teaching lab members about peer review [32], and the availability of online training modules created by journals or scientific associations. In this context, preprint servers and other online resources, for example focused on post-publication review, would facilitate the development of initial review abilities in young scientists.

Recently, Superchi et al. developed ARCADIA, a novel instrument proposed for assessing the quality of peer review reports [2], which includes 14 items in five domains (such as comments on robustness of the study and on reporting and transparency of the methods). There is a need for further validated instruments guiding the writing of peer reviews and for their assessment by editors.

It also has been identified that female scientists and researchers from upper middle income and low income countries are underrepresented as reviewers of manuscripts in international journals [6]. This highlights the need for strategies in promoting their further effective participation in the peer review process.

Regarding incentives for peer review, there has been a growing discussion about the best options for recognizing adequately the time and expertise spent on it by scientists [33]. One major aspect would be further recognition of peer review activities in promotion of scientists, by universities or national bodies. Additionally, there is the need for further discussion of monetary compensation (including discounts in article processing charges) [33], as this might create some unintended negative effects [30].

## A brief discussion of possible future developments in the peer review system

In the context of the future of biomedical publishing [34], Dr. Richard Sever has proposed recently a potential ecosystem where dissemination of manuscripts, carried out by preprint servers [35], is decoupled from evaluation, which might be done by multiple entities, such as journals, badging services or comment platforms, among others [36]. In that possible ecosystem, articles might exist only on preprint servers where they could be reviewed and commented on. It is expected that the importance of post-publication peer review will increase the following years [34].

The future of peer review would depend on the evolution of scientific manuscripts [1], with a possible growth of associated high-volume Open Data [37, 38], in addition to a potential increase in Registered Reports [39], among others. Recent advances in novel powerful tools based on artificial intelligence offer interesting opportunities to support the peer review process, such as the automated identification of manipulated images [40], among others.

There is an opportunity for journals and publishers to carry out research with the data from their submissions and reviews [6], identifying the effects of novel innovations in the peer review process. In addition, there is the need for further research in evaluating the effect of training activities related to peer review [31, 41].

## Data Availability

No datasets were generated or analysed during the current study.
